# Women’s use of online health and social media resources to make sense of their polycystic ovary syndrome (PCOS) diagnosis: a qualitative study

**DOI:** 10.1186/s12905-024-02993-5

**Published:** 2024-03-05

**Authors:** Julia Gomula, Mark Warner, Ann Blandford

**Affiliations:** Computer Science Department, UCL, Gower Street, London, UK

**Keywords:** PCOS, Polycystic ovaries syndrome, Information interaction, Finding normal, Online health communities, Sense-making, Peer support

## Abstract

**Background:**

With the growing availability of online health resources and the widespread use of social media to better understand health conditions, people are increasingly making sense of and managing their health conditions using resources beyond their health professionals and personal networks. However, where the condition is complex and poorly understood, this can involve extensive “patient work” to locate, interpret and test the information available. The overall purpose of this study was to investigate how women with polycystic ovary syndrome (PCOS) across two healthcare systems engage with online health resources and social media to better understand this complex and poorly understood lifelong endocrine disorder.

**Methods:**

A semi-structured interview study was conducted with women from the US ($N=8$) and UK ($N=7$) who had been diagnosed with PCOS within the previous five years. Transcribed data was analysed using a reflexive thematic analysis method.

**Results:**

We highlight the information needs and information-seeking strategies women use to make sense of how PCOS affects them, to gain emotional support, and to help them find an effective treatment. We also show how women with PCOS use online health and social media resources to compare themselves to women they view as “normal” and other women with PCOS, to find their sense of “normal for me” along a spectrum of this disorder.

**Conclusion:**

We draw on previous models of sense-making and finding normal for other complex and sensitive health conditions to capture the nuances of making sense of PCOS. We also discuss implications for the design and use of social media to support people managing PCOS.

**Supplementary Information:**

The online version contains supplementary material available at 10.1186/s12905-024-02993-5.

## Introduction

Polycystic ovary syndrome (PCOS) is a lifelong endocrine disorder experienced by between 6% and 13% of women [1] and is the most common endocrine disorder found in women. PCOS is a heterogeneous disorder with a spectrum of phenotypes [2–5]. The criteria for diagnosis differ [1], but are commonly menstrual irregularities, hyperandrogenism, and/or polycystic ovary morphology detected via ultrasound [3, 6]. Other symptoms that present in women vary greatly, making clinical care and research challenging [3, 6]; these include hirsutism, alopecia, acne, obesity, anxiety, depression, and stress [3, 6], as well as psycho-social impacts such as feeling “different”, struggling with notions of femininity, and wanting to be “normal” [7–9]. As well as the health complications outlined above [10], women with PCOS also have an increased risk of developing eating disorders [11], suicide [12], and sexual dysfunction [3, 11, 13].

The clinical recommendations for managing PCOS focus on targeting specific symptoms through medication and implementing lifestyle changes such as adjustments to diet and exercise [6, 14–17]. Finding the most effective medication options and lifestyle changes for PCOS can be challenging for women due to the different ways in which PCOS presents itself. As a result, there is a growing focus on women’s lived experiences with PCOS and on their information needs [18, 19]. Although information on PCOS is widely available online, it is often contradictory and of variable quality [20], lacks comprehensive, specific, and accurate details on lifestyle changes for managing PCOS symptoms [21], is not culturally specific [18, 22], and is not developed at appropriate education levels [22]. Moreover, it can be difficult to obtain reliable information from doctors and general online health resources [19, 23–27]. These shortcomings mean women can have incomplete information, limiting their ability to make effective lifestyle changes, such as to diet and exercise [21]. Little is known about how women with PCOS find or make sense of information to help them fully understand their condition and adjust their lifestyles to manage it.

Prior research has considered women’s experiences of PCOS (e.g., [9, 18, 19]), their information-needs [19, 28–30], the accuracy of PCOS information online (e.g., [31]) and women’s information-seeking behaviour relating to PCOS [28, 30]. Most prior research on information-seeking and information needs focuses on women’s practical and clinical needs - e.g., for diagnosis and treatment plans. Holbrey and Coulson [32] investigated women’s experiences of online peer support within a defined online community, and identified factors that made participants feel more or less empowered by participating in the community; they did not, however, investigate how women sought out or made sense of information.

Within the broader literature on information-seeking and sense-making related to health conditions, it has been recognised that interpersonal information-seeking allows people to engage in an information exchange [33], to share their views and lived experiences and help them make sense of health information (i.e., interpret and integrate information into their own understanding) [34, 35]. This process of information-seeking and sharing can help people to develop an understanding of what is normal for them, personally, experiencing their condition [36]. More broadly, people with long-term conditions are often concerned with feeling “normal” [37–39] and tend to compare themselves to their peers to normalise their experiences [40]. The behaviour of seeking information online to compare the personal experience of an illness to the lived experiences of peers is a recurrent theme in the literature on living with long-term conditions [41–44]. However, none of the prior literature on seeking information online to “find a new normal” has explicitly considered PCOS: either to understand women’s experiences of engaging in this kind of information-seeking online (going beyond seeking clinical and practical information) or to compare “finding normal” for PCOS with “finding normal” for other long-term health conditions.

This study aimed to investigate online information-seeking, sense-making, and “finding normal” behaviours to better understand the types of support women look for online and how support is used to help them manage their condition. It offers a new understanding of how women with PCOS manage an abundance of both clinical evidence-based information and experiential information derived from other people’s lived experiences to “find a new normal” for themselves.

## Method

### Recruitment and ethics

In developing our recruitment protocol, we were mindful of the potential impact that our research could have on participants, as well as the quality of the information we obtained through our interviews. We implemented several stages into our recruitment process to ensure freely informed consent was obtained. To allow prospective participants to learn about the study without having to reveal themselves to the research team, we published a study website that detailed information about the research, and what participants would be asked to do. The website also disclosed that the first author had been diagnosed with PCOS; this was intended to enable potential participants to anticipate her background (e.g., not a health professional) before deciding whether or not to participate in the study. As shown in the topic guide, the interviewer did not explicitly draw on her own experiences during the interviews, but this shared background may have increased rapport between interviewer and interviewee. Links to the website were posted on online PCOS support groups hosted on Facebook and Reddit. No individuals were directly approached by the research team, with participants themselves instigating contact. Once contact had been made, participants were provided with an information sheet and consent form, with guidance on how to withdraw from the study, without being disadvantaged.

To be eligible to participate in the study, participants were required to be over 18, be living in either the US or UK, and have received a formal PCOS diagnosis within the previous five years. A maximum of 5 years was chosen to increase the likelihood of participants remembering their experiences of receiving a diagnosis; and to cover a period (2014-2019) where we could assume that women would have had access to a reasonable volume of information about PCOS online; and reflecting the rise of social media use. No minimum time since diagnosis was set as this allowed us to capture insights from those who were going through this process of understanding their condition. While experiencing a diagnosis of PCOS can be distressing, our interviews focused on participants’ information practices, as opposed to their emotional journeys. Moreover, the indirect nature of our recruitment protocol meant participants were free to make their own assessment over whether they wished to participate, with the option to withdraw at any time. We included participants across two different geographical areas (the US and the UK) to obtain a broader understanding of information-seeking behaviours across different healthcare systems. The US and UK were chosen as the lead author (and interviewer) had resided in both countries and was familiar with both healthcare systems.

Participants all gave informed consent before the interview. No participants were known to any of the authors prior to the start of the study. The project was approved under UCL departmental ethics (UCLIC/1819/006/BlandfordProgrammeEthics). Participants were compensated for their time with a 15GBP (approx. 19USD) voucher.

**Table 1:** Location, time since diagnosis and age for participants included in the analysis. *Note:* all data are approximate

ID	Location	Time since diagnosis	Age
P1	US	3 years	28
P2	US	2.5 years	27
P3	US	4 years	20
P4	UK	1 month	23
P5	US	5 years	34
P6	US	1 year	31
P7	US	4 years	29
P8	UK	2.5 years	31
P9	UK	1 year	20
P10	US	4 years	28
P11	US	1.5 years	21
P12	UK	1 year	27
P13	UK	5 months	20s
P14	UK	4 years	27
P15	UK	1 year	24

### Participants

The study website attracted 507 unique visitors. Of these, 156 completed the contact form. Some were eliminated from the potential participant pool as they resided outside the US and UK. Others self-reported to have been diagnosed more than five years ago. For those that met the inclusion criteria, selection was based on whomever could schedule a mutually convenient interview time. 17 interviews were conducted between June and July 2019. However, one participant was found not to meet the inclusion criteria (she had been diagnosed more than five years ago) and for another, the recording failed. The remaining 15 women were aged between 20 and 34 (mean of 26 years), with 8 living in the US and 7 living in the UK at the time of the study. All self-reported to be living with PCOS with time since diagnosis ranging from 1 month to 5 years (mean of 28 months). Demographic information collected on the pre-interview contact form is shown in Table 1. Some participants self-reported other demographic information (e.g., profession) during interviews, which we reference in our findings where relevant.

### Procedure

Semi-structured interviews (face-to-face and online) were conducted to explore the information needs, behaviours, and technology use of women who had been diagnosed with PCOS. The interview questions were inspired by Dervin [45]’s sense-making methodology and incorporated elements of the Micro-Moment Time-Line Interview, in which interviewees are asked to consider a situation they had encountered, describe what happened, describe what questions they had, how they answered those questions, what helped or hindered them in the process, how they used those answers, and how that affected them (see: Additional files).

In the first part of the interview, participants were asked to recall how much information they had received from their doctors during their diagnosis, whether they were satisfied with that information, whether they looked for any information on their own, and what digital resources they used to do so (e.g., apps, websites, forums). Participants were then asked to reflect on their information journey before and post-diagnosis and to recall a specific example of information-seeking. The subsequent questions examined why participants chose the information resources they did and how effective they found them. In the second part of the interview, they were asked how well they felt they currently understood PCOS and their symptoms, how their information-seeking practices had changed over time, and what information sources and technologies they were currently using to help them manage their condition. Interviews lasted between 45-90 minutes, averaging approximately 1 hour.

#### Data analysis

We used the reflexive thematic analysis (RTA) approach [46, 47] to inductively analyse our data as this approach is method and theory-agnostic, meaning we were able to use RTA with a constructivist approach [48]. Joffe [49] suggests that this paradigm is well aligned with RTA as data analysis can help surface how social constructs develop. Using this approach we drew from prior research (theoretical frameworks) to help interpret our data and themes as they were developing, as opposed to deductively mapping the data to pre-existing frameworks. This approach encouraged the investigation and consideration of prior literature without forcing prior knowledge into the analysis process. Moreover, RTA allowed us to analyse our data for both semantic and latent codes and was a more accessible form of analysis for the early career researcher leading the analysis [47, 50].

Audio recordings of interviews were transcribed verbatim, omitting filler words and opening and closing formalities. In keeping with RTA [46, 47], the first author became familiar with the data while transcribing through initial memo taking to record any insights and observations. Then, the first author read the data and performed inductive open coding to develop an initial set of semantic and latent codes which were then grouped into candidate themes. To facilitate immersion, data were hand-coded. The first author reflected on the data, the codes, and themes by examining relevant theories within existing literature which allowed them to inform the themes further. Themes were reviewed and refined through discussions with the second and third authors (i.e., conceptualisation ‘checks’) who both have experience in digital health research, and particular expertise in health-related information-seeking and sense-making; however, neither have personal or professional expertise in PCOS. Finally, new themes were named, grouped further, and refined. To ensure quality practice in our analysis, we again drew from the RTA [47], and in particular from [50]; this included the thorough transcribing of audio records and checking of themes against each other and the original data and codes to ensure coherent, consistent, and distinctive themes. In keeping with RTA [51], we did not perform data saturation as the constructivist approach does not lend itself to this method. Assumptions embedded within a constructivist paradigm are that new meaning is always theoretically possible, and so defining an objective point where no new meaning can be derived is not appropriate.

## Findings

Two main themes and several sub-themes were developed from our analysis. The first main theme was information needs and participants’ strategies for finding information they required to fill a current information gap between what they knew and what they felt they needed to know. Under this theme, we explore the role of clinicians in supporting sense-making, the challenges of establishing relevance and reliability of information, and the need for experiential information in addition to evidence-based information to fill gaps and provide emotional support. The second theme was how participants redefined “normal” for themselves as they went through their PCOS information journey, how they compared themselves to “normal” women, to other women with PCOS, and how they found their “normal for me” through a process of trial and error. In presenting the themes, we incorporate several participant quotes, which we expand on in Table 1A (See: Additional files).

### Information needs and strategy

#### Pre-diagnosis: triggers for seeking information and a diagnosis

Although participants were not asked directly about their experiences prior to diagnosis, thirteen of the fifteen participants described what led them to seek a diagnosis. Two were diagnosed during a routine clinical appointment without specifically asking about it. For example, P1^US^ explained: “I was diagnosed when I was seeing a nurse practitioner just for a general checkup. She said she noticed three criteria I met”. Some participants had done a substantial amount of research, so were anticipating a PCOS diagnosis, for example, P14^UK^ said: “I was pretty sure, without a doubt about it and I had learned a lot about it up until that point. So, just being told, ‘Yeah, you have it,’ I was like, ‘OK, I kind of know everything, really, at this point,’ because it had been so long without any support or diagnosis up till that point”.

#### Lack of information from health practitioners drives independent information-seeking

Most participants were dissatisfied with the information they received from health practitioners at the time of diagnosis. Some reported receiving no information from their doctors and were instead told to search the Internet. P15^UK^ said: “I asked [my doctor] about it, she just said, ‘Look it up on the internet, there’s a lot of information on there”’. Some felt that their doctors offered them medications and oral contraceptives instead of presenting them with a broader range of treatment options, with P2^US^ saying: “they didn’t seem to inform, just to throw these medications at you and then, that’s it”. Most participants reported leaving appointments without having understood what PCOS was, and what it was going to mean for them. They felt that their health practitioner offered limited emotional support. To help address this lack of information from their health practitioner, some women turned to online social platforms for support from others with PCOS; this often helped them navigate their doctor-patient relationships. P2^US^ experienced this, saying: “A lot of women on there [Reddit] were saying how they weren’t satisfied with whatever their gynaecologist told them, and a lot of them were saying, ‘Go to a reproductive endocrinologist’. That’s what tipped me off”.

When women experienced emotionally supportive and informative conversations with medical practitioners, they reported a more positive journey following diagnosis. P11^US^ sought advice from a specialist in endocrine disease who was both supportive and informative, which empowered her to seek further information online: “She gave me all of the basic information I needed […] to start my research”. Yet for others, information received from medical practitioners had a less positive effect, with P1^US^ becoming “overwhelmed”, thinking that having PCOS was “world-ending”. For P2^US^, online research enabled her to see PCOS as a manageable condition.

#### Online medical information is seen as too general and impersonal

Participants reported initial internet searches for PCOS leading them to popular and established health information and PCOS-specific websites. While these were seen as broadly informative, they were often not specific enough to address some of the unique needs our participants had. For example, P1^US^ said: “A lot of them didn’t go into depth as to what caused certain side effects or reactions, which is what I was looking for”. Using research platforms such as Google Scholar to search for peer-reviewed articles about PCOS was a common strategy for finding information that was considered reliable and specific. Moreover, specific search strategies were discussed when using these platforms, such as applying additional filters. P8^UK^ said: “I will use Google Scholar […] and I’ll filter it by my phenotype and other potential treatment options”.

One woman (P14^UK^) described experiencing a rare symptom (Acanthosis^1^) that was not listed on the UK National Health Service (NHS) website, but was mentioned on the online social platform Reddit where “there were other people talking about it”, which helped her to understand her symptoms. For many, the use of social platforms provided a more personal experience and were considered more “real”. P3^US^ said: “I want it to be personal, not cold. Maybe medical facts and then related questions and then advice or others’ experiences”.

#### Experiential information fills gaps and offers emotional support

All participants supplemented evidence-based medical information with experiential information sources, such as social media and personal blogs. P10^US^ said: “I wanted more information, so I read all these blogs and people’s own experiences”. Online medical advice lacked the emotional and personal aspects that many women sought. P3^US^ said she wanted the “advice of people that have already been through this or know what it means”.

Women valued the more emotional nature of social media sources as it made them feel less alone and more “normal”. P1^US^ highlighted this, saying: “reading through posts [..] showed me that I wasn’t the only one going through similar thought processes”. Social platforms offered women a broader understanding of their condition. P13^UK^ said: “I’d only heard one person’s account of it [..] I just wanted to know what other people were going through and their symptoms and their stories”. These sources also had a motivating effect which P10^US^ highlighted when she said: “I feel more empowered seeing it more, especially with social media, people who kind of ‘beat it’ almost. If I see their lifestyle, I’m like, ‘Oh look, that’s where I want to get to’, and that kind of gives a little guiding light”.

Most participants were encouraged by personal stories and intimate information that online social platforms offered. However, one woman reported negative feelings towards experiential information she read. P7^US^ compared her own symptoms with others and identified those who had similar symptoms but no solutions. She said: “[these women] were all in the same boat or worse off. I can’t say that made me feel great. It seemed like what I was dealing with was mild compared to them. And none of them had even found a solution, really, so it kind of made me feel worse”.

#### Navigating an abundance of information and its relevance and reliability

The volume of information available online was often considered overwhelming, and concerns were raised as to its reliability. P3^US^ said: “there is so much information and some of it’s contradicting itself a little bit. You’ll go on one website and it’ll tell you everything that they claim like, ‘Oh, this is the holy grail, everything you’ll need to know about birth control’. You go on some other site and it’s got two other points and you’re like, ‘Well, that didn’t match up with that”’.

Information from peer-reviewed sources as well as trusted brands such as the UK NHS were considered the most trustworthy. Yet, how information was evaluated often depended on the individual reviewing it, and their background. For instance, P8^UK^ was a scientific researcher, and whilst peer-reviewed articles were typically seen as being reliable by most, she was able to identify flaws within many of the study designs.

Although social media platforms provide women with emotional support, they tend to be seen as subjective sources of information. For example, P13^UK^ said: “Facebook, obviously, you kind of take with a pinch of salt, I guess. What one person is saying is kind of true for one person”. The emergence of women presenting themselves as “PCOS specialists” on applications like Instagram was a concern to some participants. Moreover, many participants noted that they were distrustful of websites and posts that tried to commercialise PCOS advice. P8^UK^ said about Instagram: “Occasionally, you’ll see people posting on there and they’re clearly just trying to sell you something. One pill isn’t going to magically make the whole thing disappear. You have people using language like, ‘I cured my PCOS”’.

Many participants had concluded that, whilst there was an abundance of information available online, there was limited evidence-based information available about PCOS that was considered trusted, and this may contribute to misleading information being shared. Although many participants were aware that some of the information was inaccurate, they still felt informed enough to make decisions about their treatment. P10^US^ said: “I definitely feel more empowered with my diagnosis. Even though some of my knowledge may not be entirely accurate, I definitely know what works for me, even though it’s a slow and steady process.”

#### Cross-referencing experiential and medical information

Participants rarely made decisions about medications and lifestyle changes without drawing from both medical and experiential information. P2^US^ explained how she was “cross-referencing what people have to say with actual journals”. Typically, participants would first seek medical information, and then find more individually relevant information through social media. Finally, they would confirm the validity of others’ experiences against peer-reviewed articles or medical websites through focused internet searches. P1^US^ would “start off with whatever I found in Reddit and I would have a question, ‘Well, why does this work? How does this really affect different symptoms?”’ and then used “Google Scholar and try to narrow it down”. Social media often helped women seek information about topics that they may have otherwise not thought of, for example, P4^UK^ said “things like the supplements, I hadn’t thought of that on my own. I’d only thought, ‘diet’ because I’ve always been a bit sceptical about vitamins and taking things”. Whilst most of our participants cross-referenced information, not everyone compared sources: some women preferred to rely on a single source, primarily for ease and simplicity. P3^US^ stated that: “once I found Reddit, there was no need for me to narrow it down in Google because I could narrow it down and get information that I actually needed just from that one source.”

### Re-defining normal

#### Comparing self to “normal” women

All participants made references to feeling “abnormal”, “different”, and “other”. Many reported how their menstrual irregularities, hyperandrogenism, hirsutism, and/or obesity made them feel less feminine. Not feeling like a “normal” or “real” woman greatly affected their self-esteem and identities. This lack of perceived femininity caused some to question their worthiness of love with P7^US^ saying: “It made me start to question my level of femininity and I guess my worthiness of love, especially with such an aesthetic problem that I was having. It brought about some type of identity crisis”.

The realisation of their new normal caused some participants to be concerned that they would never go back to their previous normal selves and that their future paths had permanently shifted. For example, P9^UK^ reported thinking: “‘Is this forever?’ Kind of, ‘There’s something wrong with me and I won’t be able to have a normal life”’. Cultural and family expectations created additional fertility concerns for some participants, for example, P12^UK^ said that she was “from a Mexican family, so they all have four kids. [..] It would be so annoying if I can’t”.

#### Comparing self to other women with PCOS

Participants also compared themselves to other women with PCOS. Most comparisons were made against other women’s experiences reported online. Whilst prior work shows how women utilise online support for information, and emotional support [28] that they often lack from healthcare providers [27], we also found women using these resources to help them understand what was “normal” for those experiencing PCOS. As an example, P9^UK^ was asking others online: “‘Is it normal for this to happen?’ then you get a response saying, ‘Yeah, it’s normal. It’s completely fine.’ Just things you’re worried about, you can post it on there and other women will be like, ‘Yeah, it’s normal. I’ve had this’. It’s great”. This type of online information exchange can for some result in feelings of reassurance.

Some women described online communities as a “double-edged sword” in that they were simultaneously helpful and detrimental. Online communities were capable both of inspiring action and damaging self-esteem, of offering support and causing feelings of isolation, and of spreading both positivity and negativity. As an example, P4^UK^ described feeling reassured when others discussed similar issues related to their weight, yet “other people were managing it really well and I was feeling kind of ashamed that I wasn’t”. This finding supports prior work on self-tracking that has highlighted how engagement with data can result in both positive and negative experiences [53].

Our findings also highlight social divisions between groups experiencing different PCOS phenotypes, especially between those experiencing weight gain and those not. ‘Lean PCOS’ is a label commonly attached to a specific PCOS phenotype that is not associated with weight gain or obesity, whilst ‘obese PCOS’ is a label commonly associated with weight gain or obesity [54–56]. The differences in symptoms and severity of symptoms across different PCOS phenotypes often made it difficult for women to understand and sympathise with others. P3^US^ said “I don’t have the weight gain or some of the other symptoms. Then you see sometimes, on there, they’ll be like, ‘Oh, you don’t understand my struggle. No, you don’t understand my struggle”’. For those with “lean PCOS” there was pushback from some who would question the validity of their diagnosis. P11^US^ described how online members would sometimes state “Oh, lean PCOS isn’t real PCOS”, limiting the voice of this group in online forums through attempts to delegitimise them.

Knowing that people were experiencing PCOS with more severe symptoms was a source of guilt for some. Yet, it also offered positive feelings of being fortunate that their symptoms were not “the worst case” (P6^US^). In contrast, women whose symptoms seemed less manageable described feeling “jealous” and “unlucky”. P13^UK^ said “You find yourself comparing yourself to everybody and you didn’t come on there to do that […] I feel really jealous of people that can manage it and that are getting on really well with it”.

Participants recognised the heterogeneous nature of their disorder and its spectrum of phenotypes. This recognition allowed women interacting online to better place themselves in relation to other people’s experiences making PCOS feel more manageable. For example: P6^US^ said: “It just helped me make up a spectrum of the PCOS and kind of metaphorically place myself on the spectrum, which made me feel better [..] like ‘I can do this, I can possibly get pregnant if I wanted to.’ I don’t have to scare myself into this hole of, ‘I’m just this worthless human being.”’. Although some women stated that they found comparing themselves to other women unhelpful, for others it helped them realise that women with PCOS experience it differently which led to them recognising the need to find an individualised approach to managing their symptoms. P9^UK^ said: “You have to really find what works for you and essentially, that takes a lot of time to research, to try things”.

#### Finding “normal for me” through trial and error

Participants experienced a journey towards finding their “normal for me”; this involved trial and error with various medications, apps, and lifestyle changes. Many of the women interviewed had tested medications and lifestyle changes to find an individualised approach for minimising their symptoms. Whilst this trial and error journey made them feel more in control of their futures and more comfortable in their bodies, it required significant effort, especially where women reported little support from health practitioners. For example, P14^UK^ said: “I’ve tried all of the diets and the exercises and things like that, and medications over the years. I know what works for me”.

On this journey, all of our participants reported using health-tracking apps to manage their PCOS, which included apps for tracking menstruation, diet, exercise, fertility, mental health and medication. Participants talked about tracking changes in their symptoms and menstrual cycles to help them pinpoint the cause of changes. For example, P10^US^ said: “I think I correlate [my menstrual cycle] with maintaining my PCOS because the more normal I get, the less symptoms I face from PCOS, so I can clearly track that. […] I tried different diets and stuff, so I could see when things were working and when things weren’t”.

Women attributed their successful management of PCOS to their knowledge of PCOS. Through the process of being diagnosed, finding information, comparing themselves to others, and experimenting with what works for them, women were able to learn about themselves and, ultimately, were able to find their new “normal”.

## Discussion

This study extends prior work on women’s information-seeking relating to PCOS [28, 30], their need to establish what is normal for them [57], and the broader literature on health information-seeking, sense-making and finding a “new normal” based on information-seeking [36–41, 43, 44, 58, 59]. Working at the intersection of these three themes, this study has identified health information-seeking and sense-making behaviours being applied across a spectrum for PCOS. We uncover a sense-making behaviour that involves women comparing their own health experiences to that of others through online information-seeking. This allows them to develop a mental picture of the spectrum, and place themselves somewhere on it so they can contextualise their own experiences of PCOS and find their “normal for me”.

Women engaged with online PCOS communities to find similar others to help them understand whether they were “normal” within that context. Prior work has highlighted the difficulties women face when looking for relevant information around health topics, where vast amounts of information exist [60]. Our work provides insights into how women use the spectrum of PCOS to identify information that is relevant to them, amongst the vast amount of PCOS information that is broadly available. In practice, these information-seeking and sense-making practices involved women engaging in online PCOS communities to seek the experiences of those who were close to them on the PCOS spectrum, allowing them to understand what was “normal” for them. However, the differences in symptoms and their severity often made it difficult for women to connect, understand and sympathise with others. Our work highlights how women engage in an often rigorous process of sense-making to understand where they lie on the PCOS spectrum, and what treatments and lifestyle changes work for them. We also highlight the tension that women experience between being overloaded with information about their condition and identifying information that is relevant to them and reliable. Within this discussion, we first compare our broader findings to those from prior work. We then describe in more detail the spectrum-based information-seeking and sense-making behaviour that we uncover in this work and in doing so we start to unpack the tension between excessive amounts of information related to PCOS and individual relevance and reliability.

### Women’s experiences of PCOS

Our findings support existing literature on experiences of PCOS [7, 9, 16, 32, 61], in that many women questioned their femininity and whether they were “normal” because of their symptoms, turning to others with PCOS to provide context for their own experiences. As prior work has found, online peer support helped women feel less isolated, gain access to advice and information, learn to navigate their relationships with doctors and make decisions about lifestyle management and treatment [28, 57, 62], but also increased some women’s anxiety about their own health situation [32]. Nearly half of our participants experienced a delayed diagnosis, which had a negative effect on psychological and physical well-being; this finding is supported by previous studies [9, 16, 30, 63–65].

We found participants being overloaded with PCOS related information, yet we found that most women were not receiving adequate information from their doctors at the time of diagnosis which resulted in them turning to online sources such as evidence-informed websites (e.g., NHS), social media (e.g., Reddit), and blogs; this supports prior work [9, 16, 29, 30, 63–65]. However, as found by Chiu et al. [66] and others, women reported that information from PCOS-specific medical websites was too general.

Chopra et al. [57] studied the use of information technology to support people self-managing PCOS, so there is value in explicitly comparing our findings with theirs. The findings from their analysis of interviews with women with PCOS are consistent with ours in that both highlight the variability across individual experiences of PCOS and the limited understanding of the condition, in terms of both symptoms and management strategies. Hence it is a difficult condition to manage. Chopra et al. [57] focus on the requirements of technologies for self-tracking and co-management. While many of our participants also reported on the value of self-tracking (and the need for better apps, particularly for tracking menstrual cycles), co-management was not identified as a theme in our data. Whereas Chopra et al emphasise the stigma attached to PCOS, none of our participants mentioned this as an issue. However, our participants did highlight sometimes distressing divisions within the population of women managing PCOS - particularly related to the severity of symptoms and whether or not weight management was an issue (obese vs. lean PCOS).

### Information-seeking and sense-making on a spectrum

All participants in this study recognised their need for information both prior to and following diagnosis. They accessed information systems (the internet) and other people (peers with PCOS) to find information and evaluate whether it applied to them. The findings provide evidence to support Wilson’s [67] suggestion that information-seeking is collaborative and that people participate in “information exchanges”. Women in our study shared posts within online communities to support others. It can be argued that even “liking” another woman’s post is a modern-day version of an “information exchange” as liked posts are often promoted and gain more exposure.

When these findings are examined using Dervin’s [45] gaps metaphor around sense-making, the biggest gap that women experienced was a consequence of not understanding their own bodies, as women did not understand why they were experiencing their symptoms. Searching online for potential causes and being diagnosed with PCOS were the first steps in managing uncertainty [57]. As women’s knowledge of PCOS increased, so too did their understanding of the condition; this may have also contributed to increased confidence in managing the condition [62]. Gaining knowledge of the self through experimentation with treatments is consistent with research by O’Kane et al. [41] around complex long-term conditions, and by Chopra et al. [57] and Ismayilova and Sanni [25] around PCOS.

Consistent with our findings, Burgess et al. [44] found that once patients accepted their condition, they moved from a learning phase to a phase of living with their condition. In keeping with literature on long-term conditions [36–40], we found that women with PCOS are concerned with feeling “normal” and that they compare themselves to their peers to normalise their illness experience. Similarly, in line with findings from Groven and Galdas [59], people experiencing a disruption to their perceived “normal” would directly compare themselves with others.

We found information-seeking, sense-making, and finding normal being closely linked, and that uncertainty of normality acts as a catalyst for taking action and seeking information. Moreover, turning to peers to understand “normal” is essential to supplement evidence-based information. This supports O’Kane et al.’s [41] findings that evidence-based medical sources are insufficient in validating normalcy. Their participants, like ours, were not satisfied with evidence-based medical information alone, and so supplemented it with less formal information sources such as forums and blog posts. The processing of both evidence-based and experiential information allowed our participants to compare their experiences with their peers’, and to validate the normalcy of their own experiences.

**Fig. 1:**
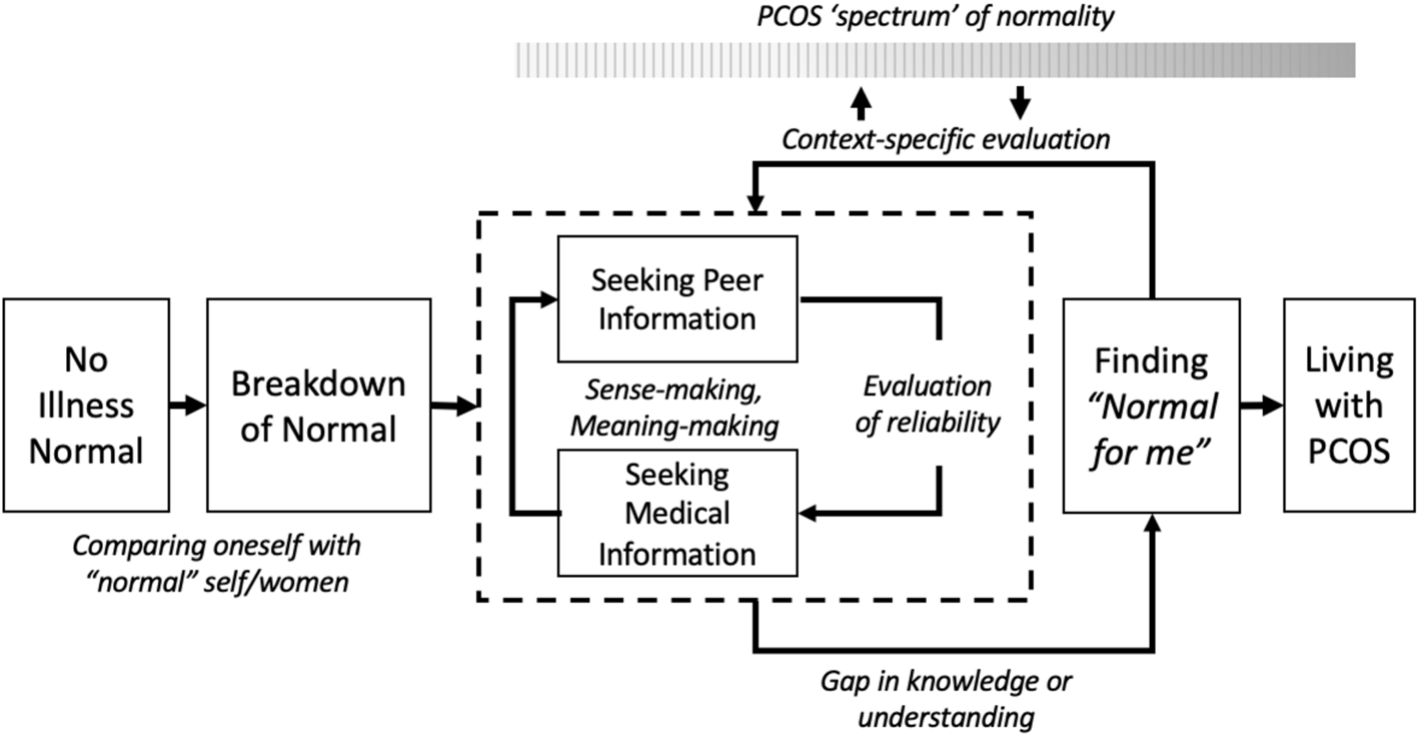
Refined model of information interaction and finding “normal for me” for PCOS

In summary, elements of our findings support those from previous studies that considered different conditions, providing evidence that those earlier findings generalise. Importantly, our study of PCOS identified and describes health information-seeking and sense-making behaviours being applied across a spectrum, and in doing so we develop a refined model (see: Fig. 1) that links together these different phases of information-seeking and sense-making.

### Finding “normal for me” for PCOS

Building on previous studies and our findings, we propose a model of information interaction and finding “normal for me” for PCOS (Fig. 1). This model is adapted from models of information interaction proposed by others for different health conditions (e.g., [36, 43]).

Several authors [68, 69] describe the initial phase of sense-making as being “life before the health condition” and note that the initial breakdown (of feeling normal) triggers information-seeking. Huttunen and Kortelainen [68] and Karp [69] describe this as just having a sense that something is not quite right. Leventhal et al.’s Common-Sense Model helps to explain these prior findings. Their model looks to understand how people respond to and manage illness threats, modelling how people use past experiences of illnesses to develop a collection of mental models of health conditions (e.g., the common cold), using these to help them identify where symptoms deviate from their usual “normative” self [70]. Within our study some participants reported similar: as they start to experience a breakdown of “normal”, they sense that something about their health is not right, although they often struggle to articulate it. We find women comparing themselves to “normal” women (including their pre-diagnosis/symptomatic selves). Women’s journeys to identifying their PCOS differ. For some, PCOS is suspected as a result of online research after experiencing symptoms. For others, the first they learn about PCOS is during their formal clinical diagnosis.

Most models identify the next important stage as seeking (or interacting with) and making sense of information about the relevant condition. Based on our analysis which found that, following diagnosis, participants generally sought out, and made what sense they could of, medical information about PCOS before turning to social media. Women begin to explore what is “normal” to experience with PCOS. Participants engage in information-seeking to make sense of their condition, which is consistent with findings from prior research in other health contexts [36, 43]. Our participants either began or continued their general PCOS search by accessing evidence-based medical websites. Supporting prior research (e.g., [36, 42, 43, 58]), we found our participants utilising both evidence-based medical information and experiential information through online social support networks, to better understand their condition. Where experiential information was thought to be unreliable, medical evidence-based information was used to check its veracity.

Genuis and Bronstein [36] and Patel et al. [43] focus on how people find personal meaning, or a “new normal” relating to their health. They differentiate between a “socially constructed normal” and an individual “new normal”, leaving it implicit that people live with that new normal. In our study, we found that seeking peer information involves understanding what is considered normal across the peer group (of people managing PCOS). However, because PCOS presents differently for each individual it is also essential to find “normal for me”, so “living with” includes self-management based on that understanding of what is normal for the individual. Thus, this sense-making process is contextually specific, with women identifying how PCOS and its symptoms vary between women, resulting in a further personalised contextualisation of information. We highlight how women with PCOS engage in sense-making to understand where they “fit” along the spectrum of PCOS by engaging with other women in online PCOS groups and reading blog posts about other women’s experiences. Determined to find their own, personal, unique “normal”, women used information from others to target their internet searches and find lifestyle changes and medications to evaluate for themselves. They tracked these changes and their results either mentally or using non-PCOS-specific health-tracking apps, which helped them gain a greater understanding of their bodies. Prior health information-seeking research has identified challenges that individuals face in efficiently identifying relevant information, despite there being vast amounts of information available [60]. In placing themselves on a spectrum of PCOS, they were better able to cope with the excessive amount of PCOS related information available to them, as women found it easier to identify what was relevant to them and their experiences with having the condition.

If their condition stabilises, women may rely less on information resources and peers, though many continue to engage with online resources and peer groups. Many also reported having adapted their lifestyles, including routinely monitoring their bodies (e.g., menstruation cycles) to manage their condition effectively over the longer term.

### Implications and further work

As highlighted in the previous section, it will be important to extend these findings to account for relevant protected characteristics such as race, culture and gender diversity. It would also be valuable to develop and test social media tools that support individuals in articulating their symptoms (“something just isn’t quite right”), identifying possible diagnoses (and the tests that would confirm them), evaluating the reliability of the information, and deciding on next steps. It would also be valuable to both test existing platforms that are designed to support people in comparing their experiences to those of others and to develop and test a novel platform that supports people in finding “normal for me” for conditions where different individuals can have significantly different symptoms and where different interventions and management strategies are most effective.

### Limitations

The external validity of this study may have been affected by recruiting participants through social media groups that were associated with PCOS. Participants recruited using these channels are likely to also use social media personally, thus skewing data towards women who already use digital tools to research or manage PCOS. However, the purpose of this study was to examine how women use digital tools and communities to seek information on and manage PCOS, not to investigate the prevalence of technology use in women with PCOS.

Our recruitment method meant that participants were self-selecting within our recruitment criteria, which resulted in a lack of homogeneity within our sample, with participants having been diagnosed with PCOS from 1 month to 5 years, and receiving clinical care across two different healthcare systems. Although the care systems in the UK and the US are substantially different, participants largely had access to the same information resources. Moreover, although the time since diagnosis differed across our sample, this allowed us to learn about information practices at different stages of people’s PCOS journey.

There are questions that, with the wisdom of hindsight, it would have been useful to address in the interviews: for example, what triggered people to start looking for information or seek a medical diagnosis? Have people explored specialised patient forums such as PatientsLikeMe, and do such forums address some of the needs they have articulated? However, our focus on information-seeking and sense-making highlighted some important needs that merit further investigation.

We did not gather information on race, culture, gender, or sexuality so are unable to add to the understanding of how these factors might influence information-seeking or - probably more importantly - the social construction of “normal” within particular cultural communities. This theme has been partially addressed by Chopra et al. [57], but merits further study.

In addition, the language used to recruit participants may have discouraged individuals with PCOS who identify as men or as non-binary from participating. These individuals may not relate to the findings of this study, especially since some findings are so closely tied to notions of femininity. Future research on PCOS should examine how this sub-population experiences PCOS.

## Conclusion

This study set out to investigate women’s information-seeking, sense-making, and “finding normal” practices when managing PCOS. Our analysis resulted in the development of two themes (1) Information Needs and Strategy and (2) Re-defining normal. Within the first theme, we describe how women use both evidence-based medical information from clinicians and online websites, as well as experiential information from online sources such as social media, forums, and blogs. They use this information to help them make decisions about potential treatments, with both types of information being necessary for women to feel that their knowledge about PCOS and their bodies is sufficiently reliable and detailed and that they are getting adequate emotional support. Within the second theme, we describe how women with PCOS seek a sense of “normal” by comparing themselves to other women whom they consider “normal” (including their pre-diagnosis/symptomatic selves) as well as to other women with PCOS. However, when they do so, they discover that PCOS is a broad-spectrum disorder that affects each woman differently. This leads them to perform a context-specific evaluation of information to help them discover what works for them as individuals so that they can find their own “normal for me”.

### Supplementary Information

**Supplementary material 1.**

## Data Availability

To protect the privacy of participants involved in this research, and the difficulties in truly anonymising our qualitative dataset, transcripts have not been made publicly available but are available on request by contacting Professor Ann Blandford.

## Footnotes

1 Acanthosis is an indicator of a rare PCOS subphenotype characterised by hyperandrogenism and insulin resistance [52]
