# Supplementary material for: Women’s use of online health and social media resources to make sense of their polycystic ovary syndrome (PCOS) diagnosis: a qualitative study
Source: BMC Womens Health. 2024 Mar 5;24:157. doi: 10.1186/s12905-024-02993-5 (PMC10913566; doi:10.1186/s12905-024-02993-5)
Supplement: Supplementary file 1 — Supplementary material 1. [file 12905_2024_2993_MOESM1_ESM.zip › Appendix table.pdf]

Appendix table

Name	Description
Table 1A	Table of representative participant quotes against the developed themes from the analysis.
Appendix 2	Semi-structured interview topic guide
