# Supplementary material for: Women’s use of online health and social media resources to make sense of their polycystic ovary syndrome (PCOS) diagnosis: a qualitative study
Source: BMC Womens Health. 2024 Mar 5;24:157. doi: 10.1186/s12905-024-02993-5 (PMC10913566; doi:10.1186/s12905-024-02993-5)
Supplement: Supplementary file 1 — Supplementary material 1. [file 12905_2024_2993_MOESM1_ESM.zip › Appendix1_Table_quotes.pdf]

Table 1A: Table of representative participant quotes against the developed themes from the analysis.

Theme	Sub-theme	Representative quote(s)
Information Needs and Strategy	Pre-diagnosis: triggers for seeking information and a diagnosis	"I went in thinking it was PCOS, had information myself, and then just went to a doctor to confirm it almost" (P6US)
	Lack of information from health practitioners drives independent information-seeking	"I have my sheet where they gave me all of the information on my ovaries, their size and stuff, and it's still so confusing. There was no explanation on it or anything, so I had to figure it out on my own." (P12UK)
	Online medical information is seen as too general and impersonal	"All the med-related content was too bland for PCOS. They just were like, 'This happens, this, this, and this'— it's like bullet points and I don't think that's enough for a person who has it." (P10US)
	Experiential information fills gaps and offers emotional support	"I just did the medical scope and then I wanted more information, so I read all these blogs and people's own experiences." (P10US)<br><br>"It was good to hear that other people were going through the same thing. Even if I wasn't talking with them directly, that helped me feel like I wasn't alone because as far as support from my family, it was my mom going, 'Oh, she can't have children,' and that was it. (P14UK)
	Navigating an abundance of information and its relevance and reliability	Occasionally, you'll see people posting on there and they're clearly just trying to sell you something. One pill isn't going to magically make the whole thing disappear.<br>You have people using language like, 'I cured my PCOS.' It's genetic." (P8UK)<br><br>"It's a Facebook group, it's a support forum for women, but also, it's a for-profit website. It's run by the person who owns the product PCOS Diet Support, all of her diets and food ingredients and stuff that she sells for people that get on her program. But other than that, she has free resources for everyone to educate themselves on PCOS and where to start." (P5US)

	Cross-referencing experiential and medical information	". . . to really do research, like to actually read multiple sources, not just take things at face value. If someone says something, it's not a fact. You have to research, you have to look at trials, look at whether a supplement actually works." (P9UK)<br><br>"It would be good if it was all in one place. I really like the idea of an app you could type questions into, and it would give you articles and things and collate things together. And yeah, a forum. [. . .] I want to make things quick and easy and at my fingertips rather than having to scroll through." (P4UK)
Re-defining normal	Comparing self to "normal" women	"With the PCOS, it's made me feel less feminine. I have four times the amount of testosterone of normal women and that concerns me." (P4UK)<br><br>"I feel like I'm not really a woman in a way, because I don't ovulate, I don't have a period, I have facial hair, my hair falls out. It really takes a hit on my self-esteem." (P12UK)
	Comparing self to other women with PCOS	"It was most helpful to read about different people having the same experiences and it helped me to feel a little bit more normal and I think that was the most important thing I got out of that, just the feeling of normalcy when all I'm seeing about myself is irregularity." (P11US)<br><br>"I just felt really bad because I don't have it as badly as other women. I felt horrible because I know the pain that I've gone through. And then, to just know that there are other women who are actually type II diabetic because of it and all of these other things, it's just sad. It almost made me hate being a woman." (P6US)
	Finding "normal for me" through trial and error	"It helped me understand different aspects of my body a bit better. [...] It helped me – I wouldn't say come to terms, but it helped me understand why [weight gain] happened. It still frustrates me to no end." (P1US)<br><br>"I think I correlate [my period] with maintaining my PCOS because the more normal I get, the less symptoms I face from PCOS, so I can clearly track that. [. . .] I tried different diets and stuff, so I could see when things were working and when things weren't." (P10US)
