# Supplementary material for: Women’s use of online health and social media resources to make sense of their polycystic ovary syndrome (PCOS) diagnosis: a qualitative study
Source: BMC Womens Health. 2024 Mar 5;24:157. doi: 10.1186/s12905-024-02993-5 (PMC10913566; doi:10.1186/s12905-024-02993-5)
Supplement: Supplementary file 1 — Supplementary material 1. [file 12905_2024_2993_MOESM1_ESM.zip › Appendix2_interview_guide.pdf]

Additional file 1 – Interview topic guide

- Greet the participant
- Remind participant of what the study is about and give a brief overview of what you'll be discussing.
- Stress: "Please don't feel pressured to discuss anything you're uncomfortable sharing with me. If you're uncomfortable with a topic, let me know, and we can move on to the next one.
- Stress anonymity and data protection

Main questions

Establishing the Situation

1) When were you first diagnosed with PCOS?
2) How much information do you remember your doctor giving you about PCOS?
    a) Were you satisfied with how much you knew about PCOS immediately after your diagnosis?

Event: Weeks following diagnosis

3) Did you look for any additional information about PCOS on your own in the weeks following your diagnosis?
    a) What tools did you use?
    b) Prompt about internet, online forums, social media communities, and apps
4) So, you received your diagnosis, you felt you needed more information, and you turned to [tools participant used] to find it. I'm going to ask you to take me through your journey as best as you can remember it. Take a few seconds to think about how you looked for information, what information you found, and how that information affected you.
    a) What questions were you facing?
    b) What thoughts did you have?
    c) What helped or facilitated you at the time, and how so?
    d) What hindered you at the time, and how so?
    e) How did this relate to your sense of self?
5) Can you think of a specific instance when you felt like you didn't know something, but maybe had to make a decision or something was on your mind? If so, what did you do to inform yourself? Please be as detailed as you can.
6) Can you tell me to what extent [tools participant used] did a good job of helping you make sense of your POS diagnosis?
    a) If discussed multiple digital tools, prompt participant until this question is answered for each specific tool.
7) What made you choose the digital tools that you did rather than [tools participant did not use?

Event: Present time

8) Now that [number of months or years] have passed since your diagnosis, how well do you feel you understand POS and your symptoms?
9) Has how you seek information about PCOS changed, and how so?
   a) Have you stopped using some tools? Started using new tools? Decreased or increased the use of tools you relied on after your diagnosis?
10) Do you use any digital tools that help you live with PCOS? Some examples could be using online support groups, a diet or exercise tracking app, a menstrual cycle tracking app, a stress management tool, a mental wellness tool, something to track symptoms, a digital diary or note-taking app, a medication tracking app, etc.? Is there anything that comes to mind?
    a) What concerns were you facing when you turned to [tool]?
    b) What thoughts did you have before using it and while using it?
    c) How did [tool] help you?
    d) What challenges did you face when using it?
    e) How did this relate to your sense of self?
11) If you think back to before your diagnosis and compare it to now, how have your understanding of your body and expectations for the future changed?

Concluding questions

12) If you could wave a magic wand and change any aspect of the digital tools you encountered in this process, what would you change?
    a) Is there a technology you wish existed either when you first learned about PCOS or to support who you are today?
13) Is there anything that you think is important for me to know, that we haven't discussed already?

- Thank participant for their time. Give compensation or explain how compensation will be sent electronically.

- Explain how results can be accessed after the study is finished.
